# How to incorporate chronic health conditions in oncologic decision-making and care for older patients with cancer? A survey among healthcare professionals

**DOI:** 10.1007/s41999-023-00919-2

**Published:** 2024-03-20

**Authors:** P. A. L. (Nelleke) Seghers, Siri Rostoft, Shane O’Hanlon, Anita O’Donovan, Karlijn Schulkes, Isacco Montroni, Johanneke E. A. Portielje, Hans Wildiers, Pierre Soubeyran, Marije E. Hamaker

**Affiliations:** 1 Department of Geriatric Medicine, Diakonessenhuis, Bosboomstraat 1, 3572 KE Utrecht, The Netherlands; 2 Department of Geriatric Medicine, Oslo University Hospital, 0424 Oslo, Norway; 3 Institute of Clinical Medicine, University of Oslo, 0318 Oslo, Norway; 4 Department of Geriatric Medicine, St Vincent’s University Hospital, Dublin, D04 T6F4 Ireland; 5 Department of Geriatric Medicine, University College Dublin, Dublin, D04 V1W8 Ireland; 6 Applied Radiation Therapy Trinity (ARTT), Discipline of Radiation Therapy, School of Medicine, Trinity St. James’s Cancer Institute, Trinity College Dublin, University of Dublin, Dublin, Ireland; 7 Department of Pulmonology, Diakonessenhuis, 3582 KE Utrecht, The Netherlands; 8 Division of Colorectal Surgery, Ospedale Santa Maria delle Croci, Viale Randi 5, 48121 Ravenna, Italy; 9 Department of Medical Oncology, Leiden University Medical Center-LUMC, 2333 ZA Leiden, The Netherlands; 10 Department of General Medical Oncology, University Hospitals Leuven, Louvain, Belgium; 11 Department of Medical Oncology, Institut Bergonié, Inserm U1312, SIRIC BRIO, Université de Bordeaux, 33076 Bordeaux, France

**Keywords:** Multimorbidity, Chronic health conditions, Older patients, Decision-making, Geriatric oncology

## Abstract

**Purpose:**

A substantial proportion of patients with cancer are older and experience multimorbidity. As the population is ageing, the management of older patients with multimorbidity including cancer will represent a significant challenge to current clinical practice.

**Methods:**

This study aimed to (1) identify which chronic health conditions may cause change in oncologic decision-making and care in older patients and (2) provide guidance on how to incorporate these in decision-making and care provision of older patients with cancer. Based on a scoping literature review, an initial list of prevalent morbidities was developed. A subsequent survey among healthcare providers involved in the care for older patients with cancer assessed which chronic health conditions were relevant and why.

**Results:**

A list of 53 chronic health conditions was developed, of which 34 were considered likely or very likely to influence decision-making or care according to the 39 healthcare professionals who responded. These conditions were further categorized into five patient profiles. From these conditions, five patient profiles were developed, namely, (1) a somatic profile consisting of cardiovascular, metabolic, and pulmonary disease, (2) a functional profile, including conditions that cause disability, dependency or a high caregiver burden, (3) a psychosocial profile, including cognitive impairment, (4) a nutritional profile also including digestive system diseases, and finally, (5) a concurrent cancer profile. All profiles were considered likely to impact decision-making with differences between treatment modalities. The impact on the care trajectory was generally considered less significant, except for patients with care dependency and psychosocial health problems.

**Conclusions:**

Chronic health conditions have various ways of influencing oncologic decision-making and the care trajectory in older adults with cancer. Understanding why specific chronic health conditions may impact the oncologic care trajectory can aid clinicians in the management of older patients with multimorbidity, including cancer.

**Supplementary Information:**

The online version contains supplementary material available at 10.1007/s41999-023-00919-2.

## Key summary points

**Aim:**

This study aimed to (1) identify which chronic health conditions may cause change in oncologic-decision-making and care in older patients and (2) provide guidance on how to incorporate these in decision-making and care provision of older patients with cancer.

**Findings:**

Thirty-four relevant health conditions were identified and subsequently combined in five profiles, consisting of conditions with similarities regarding involvement of healthcare professionals, consequences for oncologic treatment decisions, or the care trajectory. Furthermore, seven reasons related to decision-making and support or care were identified for why the presence of these profiles would influence oncologic decision-making and/or the subsequent care trajectory.

**Message:**

Assessing a patient’s health condition in light of these profiles and reasons, could aid clinicians in the management of older patients with multimorbidity, including cancer.

**Supplementary Information:**

The online version contains supplementary material available at 10.1007/s41999-023-00919-2.

## Introduction

As the population is ageing, an increasing proportion of patients with cancer are old. By 2040, 77% of patients with cancer will be older than 65 [1]. Older patients have a high prevalence of multimorbidity [2–5]. In the future, the management of older patients with multimorbidity including cancer will become a core task for cancer specialists [6]. These patients require a different approach and adaptation of current oncologic care.

Over the past decades, geriatric assessment (GA) has become the gold standard to assess older patients with cancer [7]. In a GA, multiple domains of an older patient’s health status, such as the somatic, psychological, functional and social domain, are systematically evaluated. So far, geriatric oncology research has given little attention towards the incorporation of multimorbidity into oncologic care.

In the early days of geriatric oncology, the results of the GA were simplified and used to develop fit-vulnerable-frail algorithms that decided whether patients could receive standard, adapted or no oncologic treatment. However, such simple categorizations cannot replace an overall assessment interpreted within the context of the patients and the disease [8, 9]. Tailoring oncologic care to older oncologic patients can be challenging. Geriatricians, who will all be familiar with GA and multimorbidity, may lack knowledge on how to translate its outcome to an oncologic treatment decision. Whereas, cancer specialists are often not familiar with GA and multimorbidity. Further guidance on how to incorporate chronic health conditions—both as toxicity/complication risk modifiers and as competing causes of death—in oncologic decision-making, and on how adjust care accordingly may aid these clinicians.

Previous research on multimorbidity in oncology has mainly focussed on summarizing comorbidity indexes for older patients, such as cumulative illness rating scale-geriatric (CIRS-G) and Charlson comorbidity index (CCI) [10, 11]. However, these instruments lack context for the individual and do not guide clinicians on how to incorporate chronic health conditions into oncologic care [12]. Other research addressed multimorbidity using latent class analyses to find frequently co-occurring disease-clusters [13, 14], or by clustering illness based on their impact on prognosis or quality of life domains [4, 13–15]. None of these studies were designed to cluster patients based on similar care needs or similar decision-making challenges. Thus, although the relevance of chronic health conditions in oncologic care is widely acknowledged, guidance on how to incorporate into the patient’s care pathway is lacking.

Multiple definitions of multimorbidity exist. In this study, we will define it as the co-occurrence of multiple health conditions without one holding priority [3]. Moreover, relevant geriatric impairments were included as health conditions, because comorbidities mostly cover the somatic dimension, whereas older patients may also have relevant deficits in social, psychological and functional domains [16–18]. Since we are speaking of both (comorbidities and deficits), the term “chronic health conditions” was used throughout the paper.

In this study, we gathered input from literature and geriatric oncology and geriatric experts on what chronic health conditions they consider relevant and how they use information on chronic conditions in oncologic decision-making and the subsequent care trajectory.

## Methods

This study was part of the “*Streamlined Geriatric and Oncological evaluation based on IC Technology” (GERONTE) study* (geronteproject.eu). This project aims to develop and test a new patient-centred, holistic care pathway for older patients with breast, colorectal, lung, or prostate cancer as well as other significant chronic health conditions.

The study protocol was reviewed by the ethics committee (Medical Research Ethics Committees United) and performed in accordance with the declaration of Helsinki.

### Developing the patient profiles

First, a scoping literature search was performed to identify papers that investigated the most common health problems or examined comorbidity clusters in patients with cancer (results see WebAppendix I). No specific data for older patients were found. From these studies and clinical judgement, a list of prevalent chronic health conditions in older patients was made by geriatricians MH, SR and SOH. Conditions were listed broadly, without strict definitions or subclassifications.

The next step consisted of two rounds of online surveys with an expert panel, consisting of European healthcare professionals involved in oncologic care for older patients. They were approached using purposive sampling. The following respondent data were collected: age, gender, profession, years in clinical practice, and the treatment types and cancer types they were involved in.

In the first online survey round, experts were asked to score each chronic health condition on a four-point Likert scale regarding how likely it was that (a history of) that condition would (1) change their oncologic treatment decision and (2) change the patients’ care and support needs (care trajectory). Respondents were also asked for any relevant chronic conditions that were missing from the initial list. Health conditions were carried forward to the next round if 50% of the participants scored them with likely or very likely for either one of the questions (treatment decision or care trajectory) or if both questions were answered with likely or very likely by at least 30% of the participants.

The aim of the second survey round was to further assess how these chronic health conditions could be incorporated into oncologic care. As it was considered unfeasible to ask this separately for all conditions carried forward, patient profiles were made.

The initial scoping literature review had not yielded any patient profiles that grouped conditions based on their relevance for oncology (Webappendix I). Thus, new patient profiles were designed. Conditions with similar involvement of non-oncologic healthcare professionals, or similar consequences for oncologic treatment decisions or care were grouped together by one author (MH) and subsequently refined, first by co-authors SR, SOH and NS, and afterwards based on feedback from a geriatrician, radiation oncologist, pulmonologist, surgeon and several medical oncologists.

### Relevance of patient profiles

Five patient profiles were presented to the expert panel in the second survey round; experts were asked if they agreed with the profiles or if there were relevant conditions missing and why. In addition, experts were asked to rate and describe the relevance of each patient profile for oncologic decision-making and care for the following treatment modalities: surgery, systemic therapy (including chemotherapy/targeted therapy/immunotherapy), radiation therapy, endocrine therapy and other therapy. Scores ranged from 1 (not relevant) to 4 (very relevant), and in an additional open answer field, respondents could present details from their answers.

Answers were coded using inductive coding by MH. The first round of coding consisted of open coding, and in the consecutive rounds focused coding was used. Codes were checked by NS and refined and changed as needed. Only descriptive data were used.

Finally, participants were asked which healthcare professionals should be involved in the oncologic care trajectory for each patient profile. They could choose from a list of 15 potential participants and choose if these participants should be involved for all patients, only in specific situations/profiles, or did not need to be involved.

## Results

### Identifying relevant chronic health conditions

**Fig. 1:**
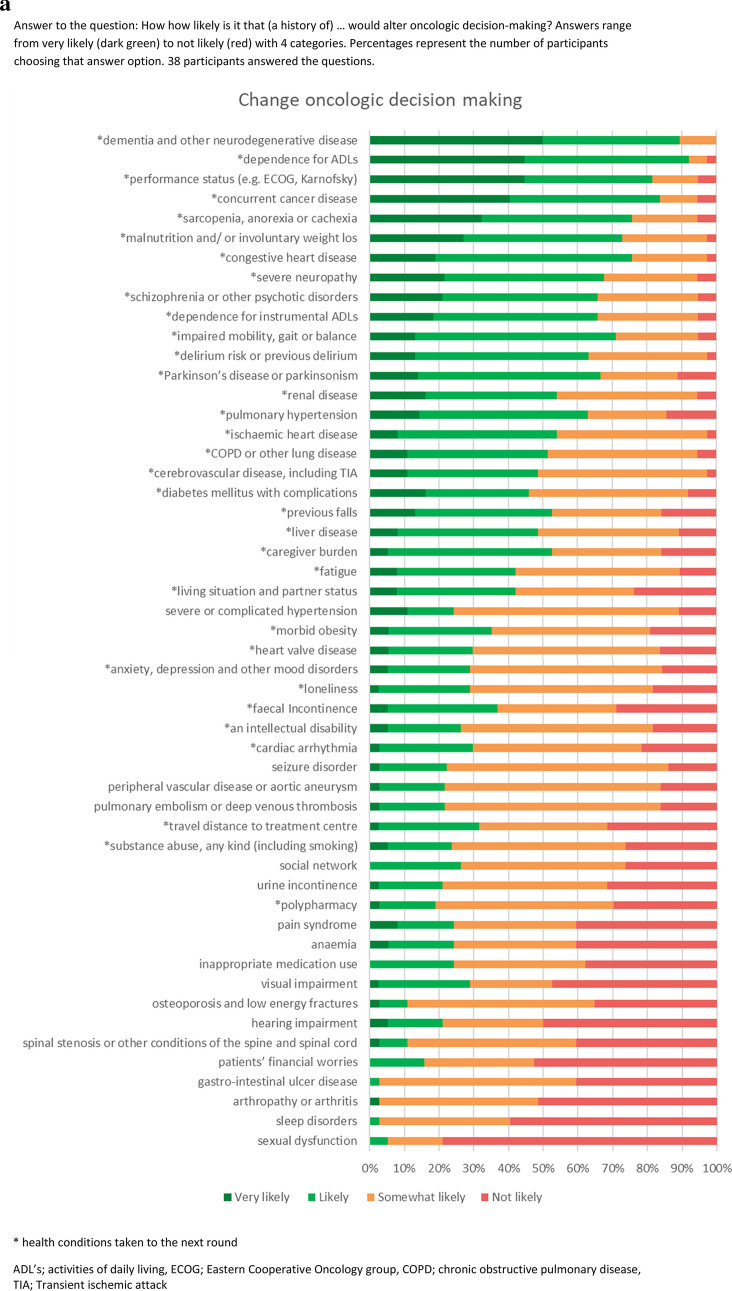

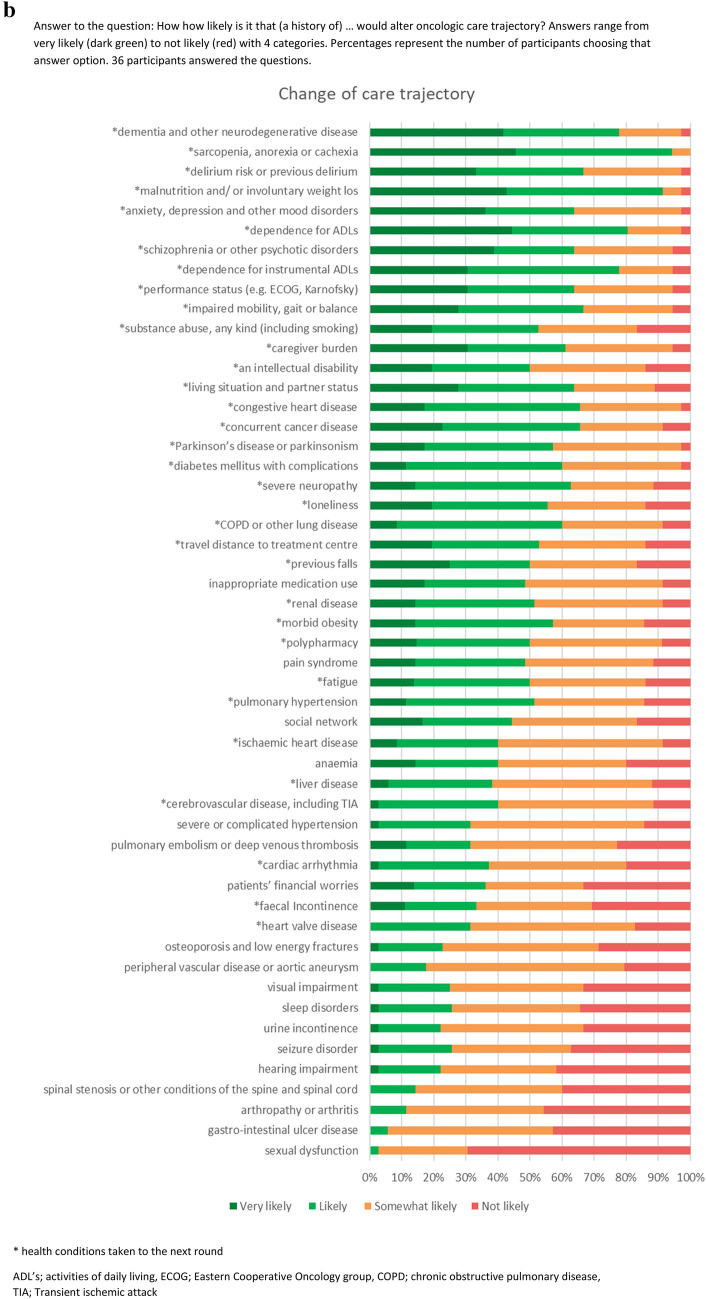
(**a**) Health conditions-likelihood to change oncologic decision-making. Answer to the question: How how likely is it that (a history of) … would alter oncologic decision-making? Answers range from very likely (*dark green*) to not likely (*red*) with 4 categories. Percentages represent the number of participants choosing that answer option. 38 participants answered the questions. (**b**) Health conditions—likelihood to change oncologic care trajectory. Answer to the question: How how likely is it that (a history of) … would alter oncologic care trajectory? Answers range from very likely (*dark green*) to not likely (*red*) with 4 categories. Percentages represent the number of participants choosing that answer option. 36 participants answered the questions. *Health conditions taken to the next round. *ADL’s* activities of daily living, *ECOG* eastern cooperative oncology group, *COPD* chronic obstructive pulmonary disease, *TIA* transient ischemic attack

In total, 53 prevalent chronic health conditions for older patients with cancer were elicited from the literature (Fig. 1a). These conditions were used in the two online expert panel survey rounds (April and May 2021). The expert panel was constructed by inviting health care professionals with expertise in geriatrics and/or oncology by email for participation. Out of 87 invited healthcare professionals, 39 agreed to participate, with a range of different specialties and backgrounds (Table 1).

**Table 1:** Demographics of the expert panel (*n* = 39)

*Healthcare professionals (years)*	
Mean age	47
Mean Years in clinical practice	17.1
*Profession (%)*	
Nurse	10
Physician	85
Researcher in Geriatric Oncology (without clinical activities)	8
*Specialty (for physicians) (%)*	
Medical oncology	30
Geriatrics	23
Other specialty …	23
Surgery	21
Organ-based specialist	10
Primary care	8
*Cancer type involved with (physicians and nurses)*^*a*^* (%)*	
Colorectal cancer	33
All cancer types	31
Breast cancer	23
Prostate cancer	21
Lung cancer	18
*Which treatments do you provide to patients yourself (physicians and nurses)?*^*a*^* (%)*	
Chemotherapy	36
Targeted and/or immune therapy	36
Hormone therapy	36
Surgery	31
None	23
Other	18
Radiation therapy	13

^a^Multiple answers per participant possible

During the first round, 18 health conditions were identified as likely or very likely to change both oncologic treatment decision-making and care trajectory, ten to only change the care trajectory, and one would only change decision-making. Five conditions did not reach >50% likely/very likely; but did reach 30–50% for both decision-making and care trajectory, and were thus carried forward (Fig. 1a, b). Based on these results, 34 health conditions were considered important to incorporate. Details can be found in Appendix 1. With regards to conditions that were considered to be missing from the initial list, only auto-immune disease was mentioned by more than one respondent.

### Developing patient profiles

Five profiles were created based on the 34 included items carried forward from Round 1, namely:

- Profile 1 Cardiovascular, metabolic and pulmonary disease—(somatic)
- Profile 2 Disability, dependency and caregiver burden—(functional)
- Profile 3 Psychosocial health problems and cognitive impairment—(psychosocial)
- Profile 4 Nutritional status and digestive system disease—(nutritional)
- Profile 5 Concurrent cancer (treatment)—(concurrent cancer)

These profiles were subsequently presented to the expert panel in the second survey round, in which 37 respondents participated. Thirty participants fully agreed with these profiles (81%). Four did not completely agree and three proposed suggestions for improvement, but since the majority agreed it was decided to keep the profiles as they were (details in Webappendix 2).

### Relevance of patient profiles

**Fig. 2:**
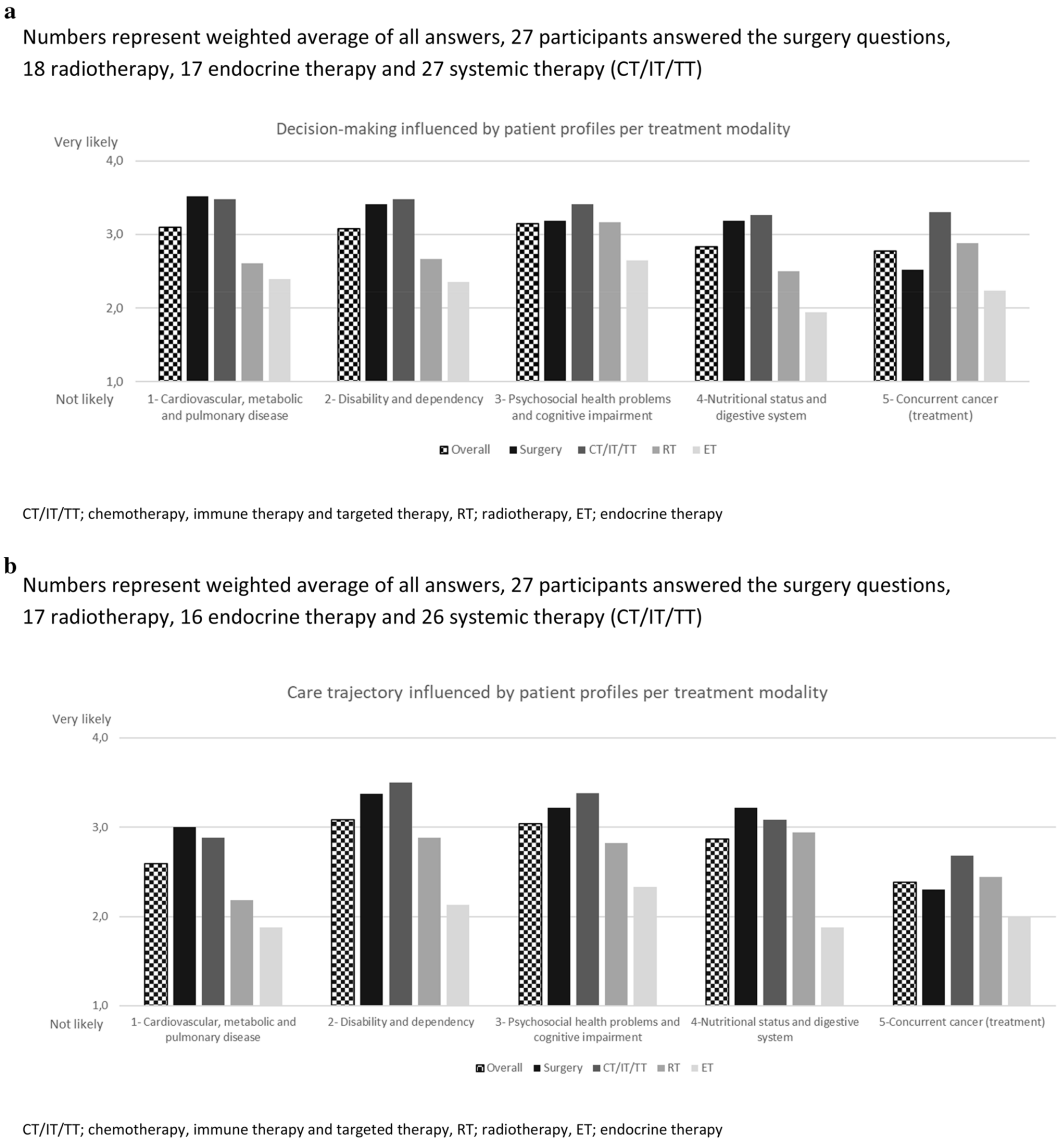
(**a**) Likelihood of the five profiles to change decision-making per treatment modality. Numbers represent weighted average of all answers, 27 participants answered the surgery questions, 18 radiotherapy, 17 endocrine therapy and 27 systemic therapy (CT/IT/TT). (**b**) Likelihood of the five profiles to change care trajectory per treatment modality. Numbers represent weighted average of all answers, 27 participants answered the surgery questions, 17 radiotherapy, 16 endocrine therapy and 26 systemic therapy (CT/IT/TT). *CT/IT/TT* chemotherapy, immune therapy and targeted therapy, *RT* radiotherapy, *ET* endocrine therapy

All five profiles had a mean relevance score of 2.8 or higher out of 4 for oncologic decision-making (Fig. 2a). In particular, the somatic, functional and psychosocial profiles were considered most likely to influence oncologic decision-making. The relevance of the patient profiles for the oncologic care trajectory was scored lower than for decision-making. Nonetheless, the functional and psychosocial profiles still received an overall score above 3, and were thus considered likely to impact the care trajectory (Fig. 2b). When assessing specific treatment modalities, decision-making and care for surgery, chemotherapy, immunotherapy and targeted therapy were more likely to be affected by chronic health conditions than radiotherapy or endocrine treatment.

Thirty respondents answered open-ended questions regarding why patient profiles would impact treatment decision-making or the care trajectory. Seven overarching reasons were formulated based on inductive coding (Table 2).

**Table 2:** Reasons why patient profiles are relevant for oncologic decision-making and oncologic care trajectory

	Reasons	1. Somatic profile (%)	2. Functional profile (%)	3. Psychosocial profile (%)	4. Nutritional profile (%)	5. Concurrent cancer profile (%)
Decision-making	Determine the feasibility of treatment and the risk of complications and toxicity	**65**	*31*	*37*	**52**	18
	Causes Interactions between cancer (treatment) and comorbidity	24	16	16	13	*47*
	Estimate resilience, impact on functional and cognitive outcomes or on quality of life	10	*34*	15	19	0
	Predict prognosis, competing causes of death	6	3	2	2	29
Care trajectory	May lead to pre-treatment optimization and need for post-treatment rehabilitation or additional discharge care	*31*	*31*	29	*44*	6
	Tailor support and care during treatment	26	*40*	**52**	19	5
Decision-making and care trajectory	Impact on decision-making capacity and lower health literacy and compliance	0	16	**55**	5	2

The italic values represent the highest values

Percentages represent participants mentioning this reason after asking why a certain profile would alter decision-making or care trajectory. Categories were made after all participants had answered the open-ended questions and were not shown to participants. Bold values represent higher percentages

Irrespective of the patient profile, three types of professionals were considered mandatory for oncologic decision-making in patients with multiple chronic health conditions: cancer specialist(s), the geriatrician and the general practitioner. Over half of respondents felt that organ-specific physicians (56%) and an oncology nurse (54%) should be involved in decision-making as well. For the care trajectory, the same specialties were identified to be important. The involvement of other (para)medical specialists should be tailored to the patient (Webappendix III).

## Discussion

This study was designed to provide an overview of chronic health conditions that may alter oncologic decision-making or care, and why. Thirty-four relevant health conditions were identified that were likely to alter oncologic decision-making an care. They were subsequently combined in five profiles, consisting of conditions with similarities regarding involvement of healthcare professionals, consequences for oncologic treatment decisions, or the care trajectory. Furthermore, seven reasons related to decision-making and support or care were identified for why the presence of these profiles would influence oncologic decision-making and/or the subsequent care trajectory. By assessing a patient’s health condition in light of these profiles and reasons, it is possible to develop a tailored, patient-centred treatment plan.

In this study, geriatric syndromes, deficits, and symptoms in other domains than the somatic domain were included as chronic health conditions in addition to more traditional diseases [18]. This was done, because these conditions may also alter the overall benefit–risk ratio of treatment and may thus lead to a different treatment decision. For example, because they are associated with increased treatment risk such as dependency for ADLs and the unavailability of someone to take them to the doctor [19]. Moreover, in case of similar benefits of treatment, a patient who lives far away may chose the treatment that requires the least hospital visits. Traditional diseases are important to take into account, but also other conditions may be determinative in the treatment choice.

The most frequently mentioned reason to alter decision-making was the effect of a chronic health condition on the feasibility of treatment and complication risks. To determine whether impact on feasibility is expected from conditions and whether this may impact treatment decision or care provision, a clinician may discuss these questions with the medical team within the context of the patients, including all treatment options and their effect on various outcomes, such as survival, functional outcomes and quality of life. The answer to this question may shift the balance between risk and benefit and could thus alter the treatment recommendation. Of note, identified deficits should not automatically lead to decline of treatment, as some conditions may benefit from optimization or additional support after which patients are still able to undergo oncologic treatment [20].

Examples of the above-mentioned are pre-treatment optimization, post-treatment rehabilitation, additional discharge care or additional support during treatment. For example, prior to surgery, physical prehabilitation may be an option to improve functional outcomes [7, 20–23]. For psychosocial health problems, the impact on decision-making capacity, self-management capacity, and compliance should be considered [21–23] and caregiver support and increased monitoring may be needed [22, 23]. Previous research can be used to identify supportive interventions to optimize the patients’ overall health to improve feasibility of treatment in vulnerable patients and thus make treatment more accessible for these patients [22, 23]. These interventions should be part of the overall oncologic treatment.

Thus, the developed profiles may help to guide the questions in relation to the chronic health conditions that are identified from the patient’s medical history or geriatric assessment. Nonetheless, these profiles cannot be simplified to easy yes-or-no/one-size-fits-all algorithms, since older patients with multimorbidity including cancer are a heterogeneous group. Moreover, the impact will not merely depend on the presence of a condition, but also on its severity, impact on daily life, and on the combination of chronic conditions in the same patient [24]. Combining conditions into profiles may also cause a risk of concealing interactions in a more broad way, for example, between other diseases and treatments that are not within one profile [25]. Therefore, the profiles should not be used to dictate treatment or care but rather as suggestions of questions that need to be elucidated before making treatment decisions.

This study has some limitations. First, due to the pragmatic method to identify potentially relevant and prevalent chronic health conditions in older patients with cancer, some conditions may have been missed. The expert panel found only one condition to be missing, and the conditions that were ultimately selected by our expert panel are similar to the selections made in studies operationalizing multimorbidity [24, 26]. Second, this study tried to identify relevant chronic health conditions for a heterogeneous group of patients, including four cancer types with all stages and different treatment modalities. Some nuances may have been lost by not considering all the details of the cancer type, stage of disease, or treatment regimens. However, with an ever-evolving scope of treatment options, such detailed guidance may be difficult to provide and could soon become obsolete. Therefore, we chose to provide guidance based on a broad classification that can be used in a wide range of situations and thus will be generalizable and remain useful in the care for the heterogenous older population. Finally, this study was not designed to look at exact percentages or differences between profiles, but to identify how chronic health conditions could impact oncologic decision-making or care. Caution is thus needed when interpreting the reported percentages. Low percentages do not necessarily mean that these reasons are less important, but only mean that the answer was given less frequently. Participants were not given the opportunity to check if reasons mentioned by others were equally or even more important to them.

Previous studies have shown that performing assessments without interventions will have little impact on clinical outcomes [6]. Therefore, this study aimed to assist clinicians in utilizing the information about chronic health conditions from medical history and geriatric assessment in oncologic care. Questions relating to the reasons mentioned in Table 2 are complex and require expertise and collaboration from various disciplines. Ideally, a multidisciplinary team discussion takes place to discuss how chronic health conditions should be incorporated into the oncologic care pathway.

In conclusion, chronic health conditions have various ways of influencing oncologic decision-making and may impact oncologic care differently throughout the care trajectory. Knowing which conditions may impact the oncologic care trajectory at what stages by asking the right questions may improve the management of older patients with cancer. Nevertheless, treatment recommendations and subsequent management of this complex population require a patient-centred and multidisciplinary approach.

## Supplementary Information

Below is the link to the electronic supplementary material.

Supplementary file1 (PDF 326 KB)

## Appendix 1: Likelihood of health conditions to change treatment decision-making or care trajectory

Percentages represent the proportion of participants stating that the health condition would likely or very likely change the treatment decision or the care trajectory. Dark colour represents the 34 conditions that were forwarded to the next round.

**Table:**

	Treatment decision (%)	Care trajectory (%)
… dependence for ADLs	92	81
… dementia and other neurodegenerative disease	89	78
… concurrent cancer disease	84	66
… performance status (e.g. ECOG, Karnofsky)	82	64
… congestive heart disease	76	66
… sarcopenia, anorexia or cachexia	76	94
… malnutrition and/or involuntary weight loss	73	91
… impaired mobility, gait or balance	71	67
… severe neuropathy	68	63
… Parkinson’s disease or parkinsonism	67	57
… schizophrenia or other psychotic disorders	66	64
… dependence for instrumental ADLs	66	78
… delirium risk or previous delirium	63	67
… pulmonary hypertension	63	51
… ischaemic heart disease	54	40
… renal disease	54	51
… previous falls	53	50
… caregiver burden	53	61
… COPD or other lung disease	51	60
… cerebrovascular disease, including TIA	49	40
… liver disease	49	38
… diabetes mellitus with complications	46	60
… fatigue	42	50
… living situation and partner status	42	64
… faecal Incontinence	37	33
… morbid obesity	35	57
… travel distance to treatment centre	32	53
… cardiac arrhythmia	30	37
… heart valve disease	30	31
… anxiety, depression and other mood disorders	29	64
… visual impairment	29	25
… loneliness	29	56
… an intellectual disability	26	50
… social network	26	44
… severe or complicated hypertension	24	31
… pain syndrome	24	49
… anaemia	24	40
… inappropriate medication use	24	49
… substance abuse, any kind (including smoking)	24	53
… seizure disorder	22	26
… pulmonary embolism or deep venous thrombosis	22	31
… peripheral vascular disease or aortic aneurysm	22	18
… hearing impairment	21	22
… urine incontinence	21	22
… polypharmacy	19	50
… patients’ financial worries	16	36
… spinal stenosis or other conditions of the spine and spinal cord	11	14
… osteoporosis and low energy fractures	11	23
… sexual dysfunction	5	3
… gastro-intestinal ulcer disease	3	6
… arthropathy or arthritis	3	11
… sleep disorders	3	26

## Appendix 2: Reasons why patient profiles are relevant for decision-making and care trajectory per patient profile

Percentages represent participants mentioning this reason. Darker colours represent higher percentages. Details on the 5 profiles are shown in Webappendix II.

**Table:**

	Determine the feasibility of treatment and the risk of complications and toxicity (%)	Predict prognosis, competing causes of death (%)	Causes Interactions between cancer (treatment) and comorbidity (%)	Estimate resilience, impact on functional and cognitive outcomes or on quality of life of life (%)	Impact on decision-making capacity and lower health literacy and compliance (%)	May lead to pre-treatment optimization and need for post-treatment rehabilitation or additional discharge care (%)	Tailor support and care during treatment (%)
***PROFILE 1**** Cardiovascular, metabolic and pulmonary disease—(somatic)*							
Surgery	**91**	14	0	23	0	**55**	*45*
Chemotherapy	**71**	0	*39*	4	0	17	22
Radiotherapy	**50**	9	27	0	0	18	0
Endocrine therapy	*33*	0	*33*	0	0	11	11
***PROFILE 2**** Disability, dependency and caregiver burden—(functional)*							
Surgery	27	9	0	**64**	0	**50**	*41*
Chemotherapy	*47*	0	*39*	32	0	17	*32*
Radiotherapy	*42*	0	17	0	25	17	**50**
Endocrine therapy	0	0	25	0	**50**	0	*38*
***PROFILE 3**** Psychosocial health problems and cognitive impairment—(psychosocial)*							
Surgery	**59**	5	0	*36*	*36*	*32*	**64**
Chemotherapy	28	0	20	0	**70**	25	**50**
Radiotherapy	*42*	0	21	7	**57**	21	21
Endocrine therapy	0	0	27	0	*36*	27	*45*
***PROFILE 4**** Nutritional status and digestive system disease—(nutritional)*							
Chemotherapy	**76**	0	18	0	**6**	*47*	18
Radiotherapy	*42*	0	23	8	0	23	*31*
Endocrine therapy	13	0	22	0	22	11	22
***PROFILE 5**** Concurrent cancer (treatment)—(concurrent cancer)*							
Surgery	19	**62**	**52**	0	0	10	5
Chemotherapy	**50**	0	0	0	0	0	**50**
Radiotherapy	*33*	13	**50**	0	0	13	0
Endocrine therapy	*33*	*33*	0	0	0	0	0

## Appendix 3: Reasons why patient profiles are relevant for decision-making and care trajectory per treatment modality

Percentages represent participants mentioning this reason. Darker colours are higher percentages.

**Table:**

	Determine the feasibility of treatment and the risk of complications and toxicity (%)	Predict prognosis, competing causes of death (%)	Causes Interactions between cancer (treatment) and comorbidity (%)	Estimate resilience, impact on functional and cognitive outcomes or on quality of life of life (%)	Impact on decision-making capacity and lower health literacy and compliance (%)	May lead to pre-treatment optimization and need for post-treatment rehabilitation or additional discharge care (%)	Tailor support and care during treatment (%)
*Surgery*							
Profile 1	**91**	14	0	23	0	**55**	*45*
Profile 2	27	9	0	**64**	0	**50**	*41*
Profile 3	**59**	5	0	*36*	*36*	*32*	**64**
Profile 4	**59**	5	0	**50**	0	**68**	14
Profile 5	19	**62**	**52**	0	0	10	5
*Chemo*							
Profile 1	**71**	0	*39*	4	0	17	22
Profile 2	*47*	0	27	*32*	14	27	*32*
Profile 3	28	0	20	0	**70**	25	**50**
Profile 4	**76**	0	18	0	6	*47*	18
Profile 5	19	29	**93**	0	7	7	14
*RT*							
Profile 1	**50**	9	27	0	0	18	0
Profile 2	*42*	0	17	0	25	17	**50**
Profile 3	*42*	0	21	7	**57**	21	21
Profile 4	*42*	0	23	8	0	23	*31*
Profile 5	*33*	13	**50**	0	0	13	0
*ET*							
Profile 1	*33*	0	*33*	0	0	11	11
Profile 2	0	0	25	0	**50**	0	*38*
Profile 3	0	0	27	0	*36*	27	*45*
Profile 4	13	0	22	0	22	11	22
Profile 5	0	0	20	0	0	0	0

Profile 1 Cardiovascular, metabolic and pulmonary disease—(Somatic)

Profile 2 Disability, dependency and caregiver burden—(functional)

Profile 3 Psychosocial health problems and cognitive impairment—(psychosocial)

Profile 4 Nutritional status and digestive system disease—(nutritional)

Profile 5 Concurrent cancer (treatment)—(concurrent cancer)
