# Supplementary material for: How to incorporate chronic health conditions in oncologic decision-making and care for older patients with cancer? A survey among healthcare professionals
Source: Eur Geriatr Med. 2024 Mar 20;15(4):1055–67. doi: 10.1007/s41999-023-00919-2 (PMC11377634; doi:10.1007/s41999-023-00919-2)
Supplement: Supplementary file 1 — Supplementary file1 (PDF 326 KB) [file 41999_2023_919_MOESM1_ESM.pdf]

Web appendix I - Papers assessed in multimorbidity literature review

A. Classifications based on types of comorbidities and most common health problems in patients with cancer

* Karen Barnett, Stewart W Mercer, Michael Norbury, Graham Watt, Sally Wyke, Bruce Guthrie. Epidemiology of multimorbidity and implications for health care, research, and medical education: a cross-sectional study. Lancet 2012 Jul 7;380(9836):37-43.

* Helen Fowler, Aurelien Belot, Libby Ellis, Camille Maringe, Miguel Angel Luque-Fernandez, Edmund Njeru Njagi, Neal Navani, Diana Sarfati, Bernard Rachet. Comorbidity prevalence among cancer patients: a population-based cohort study of four cancers. BMC Cancer 2020 Jan 28;20(1):2.

* Diana Sarfati, Jason Gurney, Bee Teng Lim, Nasser Bagheri, Andrew Simpson, Jonathan Koea, Elizabeth Dennett. Identifying important comorbidity among cancer populations using administrative data: Prevalence and impact on survival. Asia Pac J Clin Oncol 2016 Mar;12(1):e47-56.

* Grant R Williams, Amy Mackenzie, Allison Magnuson, Rebecca Olin, Andrew Chapman, Supriya Mohile, Heather Allore, Mark R Somerfield, Valerie Targia, Martine Extermann, Harvey Jay Cohen, Arti Hurria, Holly Holmes. Comorbidity in older adults with cancer. J Geriatr Oncol 2016 Jul;7(4):249-57.

B. Classifications based on types of comorbidities and most common health problems in patients in general

* Mercedes Clerencia-Sierra, Amaia Calderón-Larrañaga, et al. Multimorbidity Patterns in Hospitalized Older Patients: Associations among Chronic Diseases and Geriatric Syndromes. PLoS One. 2015 Jul 24;10(7):e0132909.

C. Classifications based on comorbidity clusters in patients with cancer

* Erin E Hahn, Michael K Gould, Corrine E Munoz-Plaza, Janet S Lee, Carla Parry, Ernest Shen Understanding Comorbidity Profiles and Their Effect on Treatment and Survival in Patients With Colorectal Cancer. J Natl Compr Canc Netw 2018 Jan;16(1):23-34.

* Michael K Gould, Corrine E Munoz-Plaza, Erin E Hahn, Janet S Lee, Carly Parry, Ernest Shen. Comorbidity Profiles and Their Effect on Treatment Selection and Survival among Patients with Lung Cancer. Ann Am Thorac Soc 2017 Oct;14(10):1571-1580.

* Kelly M Kenzik, Erin E Kent, Michelle Y Martin, Smita Bhatia, Maria Pisu. Chronic condition clusters and functional impairment in older cancer survivors: a population-based study. J Cancer Surviv 2016 Dec;10(6):1096-1103.

D. Classifications based on comorbidity clusters in patients in general

* Mercedes Clerencia-Sierra, Amaia Calderón-Larrañaga, Nicolás Martínez-Velilla, Itziar Vergara-Mitxeltorena, Pablo Aldaz-Herce, Beatriz Poblador-Plou, Mónica Machón-Sobrado, Nerea Egüés-Olazabal, Gabor Abellán-van Kan, Alexandra Prados-Torres. Multimorbidity Patterns in Hospitalized Older Patients: Associations among Chronic Diseases and Geriatric Syndromes. PLoS One. 2015 Jul 24;10(7):e0132909

* Beatriz Poblador-Plou, Marjan van den Akker, Rein Vos, Amaia Calderón-Larrañaga, Job Metsemakers, Alexandra Prados-Torres. Similar multimorbidity patterns in primary care patients from two European regions: results of a factor analysis. PLoS One 2014 Jun 23;9(6):e100375.

* Alexandra Prados-Torres, Amaia Calderón-Larrañaga, Jorge Hancco-Saavedra, Beatriz Poblador-Plou, Marjan van den Akker. Multimorbidity patterns: a systematic review. J Clin Epidemiol 2014 Mar;67(3):254-66.

* Sanne Pagh Møller, Bjarne Laursen, Caroline Klint Johannesen, Janne S Tolstrup, Stine Schramm. Patterns of multimorbidity and demographic profile of latent classes in a Danish population-A register-based study. PLoS One 2020 Aug 11;15(8):e0237375

* Ljoudmila Busija, Karen Lim, Cassandra Szoeke, Kerrie M Sanders, Marita P McCabe. Do replicable profiles of multimorbidity exist? Systematic review and synthesis. Eur J Epidemiol 2019 Nov;34(11):1025-1053.

E. Classifications based on care needs and/or functioning in patients in general

* Mieke Rijken, Iris van der Heide. Identifying subgroups of persons with multimorbidity based on their needs for care and support. BMC Fam Pract 2019 Dec 27;20(1):179.

Webappendix II – Patient profiles

	1	2	3	4	5
Name	Cardiovascular, metabolic and pulmonary disease	Disability and dependency *	Psychosocial health and cognitive impairment	Nutritional status and digestive system	Concurrent cancer (treatment)
Included items from round 1	… congestive heart disease<br>… pulmonary hypertension<br>… ischaemic heart disease<br>… renal disease<br>… COPD or other lung disease<br>… cerebrovascular disease, including TIA<br>… diabetes mellitus with complications<br>… morbid obesity<br>… cardiac arrhythmia<br>… heart valve disease	… severe neuropathy<br>… Parkinson's disease or parkinsonism<br>… dependence for ADLs<br>… performance status (e.g. ECOG, Karnofsky)<br>… impaired mobility, gait or balance<br>… dependence for instrumental ADLs<br>… previous falls<br>… fatigue<br>… faecal Incontinence<br>… caregiver burden<br>… living situation and partner status<br>… travel distance to treatment centre	… dementia and other neurodegenerative disease<br>… schizophrenia or other psychotic disorders<br>… delirium risk or previous delirium<br>… anxiety, depression and other mood disorders<br>… substance abuse, any kind (including smoking)<br>… loneliness<br>… an intellectual disability	… sarcopenia, anorexia or cachexia<br>… malnutrition and/ or involuntary weight loss<br>… liver disease	… concurrent cancer disease
New		Rheumatologic disease *			

We decided to combine impairments on social domain and functional domain, since they both lead to similar dependency and involvement of a caregiver. We also included the comorbidities that easily lead to impaired mobility here.
Polypharmacy was considered important for care trajectory in 50%, but as it may be overarching, depending on the type of medication used it was not added to one of the profiles, but considered relevant for all profiles and relevant to assess.

*Auto immune disease was given as missing item, it does not fit in one of these profiles. Since we are making common profiles, we did however add rheumatologic diseases (including Rheumatoid arthritis and polymyalgia rheumatica) in the "disability and dependency" group. Chrohn's or other chronic auto-immune colitis could be added to the digestive system if present.

Webappendix III- Involved healthcare professionals

Which of the following health care professionals should be involved in the care trajectory of older patients with multimorbidity receiving treatment for cancer? And when should they be involved?

	In all patients		No involvement		Only in specific profile		Total
	%	n	%	n	%	n	n
Oncology specialist(s) (including surgeons, radion oncologists and medical oncologists)	89%	25	0%	0	11%	3	28
Geriatrician	52%	14	0%	0	48%	13	27
General practitioner	96%	27	0%	0	4%	1	28
Oncology nurse	79%	22	0%	0	21%	6	28
Palliative care specialist	7%	2	0%	0	93%	26	28
Other organ-specific physician	0%	0	4%	1	96%	27	28
Anaesthesiologist	0%	0	29%	8	71%	20	28
Pharmacist	41%	11	4%	1	56%	15	27
Psychologist/psychiatrist	11%	3	0%	0	89%	24	27
Physiotherapist	36%	10	0%	0	64%	18	28
Dietician	30%	8	0%	0	70%	19	27
Occupational therapist	22%	6	0%	0	78%	21	27
Social worker	41%	11	0%	0	59%	16	27
Home care nurse/staff	29%	8	0%	0	71%	20	28
Clerics (or spiritual helper)	4%	1	7%	2	89%	24	27

Do you think the following professionals have a role in the initial decision-making process?

	Yes		No		Total answers
	%	n	%	n	n
Oncology specialist(s) (including surgeons, radiation oncologists and medical oncologists)	100%	28	0%	0	28
Geriatrician	100%	27	0%	0	27
General practitioner	82%	23	18%	5	28
Oncology nurse	54%	15	46%	13	28
Palliative care specialist	29%	8	71%	20	28
Other organ-specific physician	56%	15	44%	12	27
Anaesthesiologist	37%	7	63%	12	19
Pharmacist	12%	3	88%	23	26
Psychologist/psychiatrist	19%	5	81%	22	27
Physiotherapist	4%	1	96%	27	28
Dietician	7%	2	93%	25	27
Occupational therapist	0%	0	100%	27	27
Social worker	15%	4	85%	23	27
Home care nurse/staff	11%	3	89%	25	28

Clerics (or spiritual helper)	8%	2	92%	23	25
